# Impact of politeness and performance quality of android robots on future interaction decisions: a conversational design perspective

**DOI:** 10.3389/frobt.2024.1393456

**Published:** 2024-05-28

**Authors:** Waka Saeki, Yoshiyuki Ueda

**Affiliations:** ^1^ Graduate School of Education, Kyoto University, Kyoto, Japan; ^2^ Institute for the Future of Human Society, Kyoto University, Kyoto, Japan

**Keywords:** minor failure, politeness, robot design, robot impression, robot interaction, success

## Abstract

Despite robots being applied in various situations of modern society, some people avoid them or do not feel comfortable interacting with them. Designs that allow robots to interact appropriately with people will make a positive impression on them resulting in a better evaluation of robots, which will solve this problem. To establish such a design, this study conducted two scenario-based experiments focusing on the politeness of the robot’s conversation and behavior, and examined the impressions caused when the robot succeeds or slightly fails at a task. These two experiments revealed that regardless of whether the partner is a robot or a human, politeness not only affected the impression of interaction but also the expectations for better task results on the next occasion. Although the effect of politeness on preference toward robot agents was smaller than those toward human agents when agents failed a task, people were more likely to interact with polite robots and human agents again because they thought that they would not fail the next time. This study revealed that politeness motivates people to interact with robots repeatedly even if they make minor mistakes, suggesting that the politeness design is important for encouraging human-robot interaction.

## 1 Introduction

Recent advances in robot technology have resulted in robots being used in various situations such as at hotel reception desks and to serve meals where communication with people is necessary. However, people do not always accept or interact effectively with communicative robots (Urakami and Sutthithatip, 2021). This is a problem that needs to be solved to achieve a society in which humans coexist with robots by taking advantage of the benefits they offer. This study aims to investigate the kind of robot design that would encourage people to accept robots and accelerate human-robot interaction in situations where communication is desired.

Robot impression is formed by the appearance and verbal and attitudinal cues that robots exhibit (e.g., Urakami and Sutthithatip, 2021; Liberman-Pincu et al., 2023). Although these features are important in the design of a comforting robot, especially for interaction with humans, spoken and behavioral indications are equally important because they help in information transfer (Saeki and Ueda, 2024). For example, Edwards et al. (2019) revealed that the interaction method of robots based on human-human interaction increases the positive impression of the robot. In addition, both robot and human helpers are positively evaluated when they often use hedges (i.e., “I think,” “probably”) or discourse markers (i.e., “I mean,” “so”) in communication to reduce the forcefulness of advice than when they do not use them (Torrey et al., 2013). Therefore, since impressions of interaction with robots are very similar to those with humans, robot interaction design referring to human-human interactions may be able to ensure more positive impressions.

Politeness is an important cue that makes an impression in social interactions (Torrey et al., 2013; Hayes and Miller, 2016; Meyer et al., 2016). It refers to the extent to which service agents are perceived as considerate and trustworthy (Brown and Swartz, 1989) and, specifically, has a significant impact on initial trust (Coulter and Coulter, 2002). Humans as well as robots and chatbots have pointed out that politeness is comforting, and studies have investigated effective ways to use politeness (e.g., Castro-González et al., 2016; Jucks et al., 2018; Kaiser et al., 2019; Lee et al., 2019; Miyamoto et al., 2019; Smith et al., 2022). Particularly in human robot collaboration, the politest robots receive higher evaluations on enjoyment, satisfaction, and trustworthiness, regardless of participants’ age (Kumar et al., 2022). Reviewing previous studies focusing on politeness revealed that the degree of people’s willingness to follow robots’ instructions may differ between situations where people receive general services that do not require a license based on the agent’s professional ability to work (i.e., service situations; robot guards performing access-control at a building entrance, Inbar and Meyer, 2019) and situations where they receive services requiring a license, called an occupational licensing, which guarantees that the agent is qualified to perform a particular job (i.e., expert situations; a healthcare robot who works to improve mental health, Lee et al., 2017). In a service situation, when the robot greeted people at the beginning of a conversation, they considered that robot to be more polite than one that did not greet them. The impolite robot guard gave them feelings of more intimidation, less fairness, less friendliness, and less appropriateness (Inbar and Meyer, 2019). In an expert situation, Lee et al. (2017) examined whether the politeness of the robot could affect patients’ intention to comply with its requests. Participants interacted with the robot, and the results revealed that the robot’s polite behaviors decreased the perceived cost of noncompliance with the request, leading to less intention to comply. These results were consistent with those of studies on human physicians, which showed that politeness did not always make patients follow the physicians’ requests (Sinha et al., 1996). These suggest that, depending on specific situations, politeness design may not always be necessary. However, these studies did not investigate whether people prefer to interact again with less polite robots. In other words, if further interactions with less polite robots are also preferred and are effective in building relationships in expert situations, politeness design appropriate to situations may influence the formation of positive impressions and robot design that are beneficial various situations. It may encourage people to accept social robots.

In addition, the impressions of robots are not always the same as those of humans, with robots being likely to be less responsible than humans when both make similar mistakes (Leo and Huh, 2020; Furlough et al., 2021). Leo and Huh (2020) investigated which of the two, among robots and humans, was more responsible they made similar mistakes in pharmacist prescribing and the restaurant food serving tasks using a scenario-based experiment. They found that participants attributed less responsibility to the robot than to the human regardless of their experience (i.e., the degree of familiarity with, experience of using, and amount of knowledge about the service robot). Moreover, the degrees of responsibility of the agents were mediated by their controllability, which indicated the extent to which the failure was under the service agent’s control. Furlough et al. (2021) examined which of the following—humans, autonomous robots, nonautonomous robots, or the environment (e.g., a loose table leg)—were to blame when a task by a human-robot team failed in military, surgical, and warehouse scenarios. The results revealed that, in general, when a task failed, people assigned more responsibility to human than to environmental factors. When the robot was nonautonomous—performing tasks with explicit human control—people attributed less responsibility to the robot than to humans, but when the robot was autonomous—performing tasks without human control—people attributed almost as much responsibility to the robot as to humans.

From these perspectives, we should examine how to design interactions with robots to facilitate their acceptance by people into society. In service situations, both polite robots and humans gave positive impressions (Inbar and Meyer, 2019), while in expert situations, people tended to comply with requests of less polite robots and humans (Lee et al., 2017). In addition, the responsibility for task failure was attributed to the human rather than to the robot (Leo and Huh, 2020; Furlough et al., 2021). If a polite robot is perceived as a more human-like agent, the task failure responsibility attributed to the robot may be as large as to the person, because expectations of the robot’s abilities are high. Moreover, impressions and responsibility of the robot may vary depending on the type of service (service vs. expert) or their performance (success vs. failure). Thus, this study investigated the impact of robot politeness on impressions during successful/failed tasks in service and expert situations.

In Experiment 1, we aimed to investigate the effects of politeness and service type on robots’ and humans’ preference, future interaction motivations, and impressions of their contribution to achieving a task goal. In Experiment 2, we examined the belief of participants concerning the replicability and expectations for better results from polite/impolite robots’ and humans’ assistance. Both experiments were scenario-based, enabling us to manipulate the politeness of agent, agent type, and service type independently. Moreover, in service failure research, scenario-base experiments are often employed since they can avoid the cost and ethical issues of real experiments (Bitner et al., 1990), and prevent participants from experiencing real failure (Smith and Bolton, 1998; Smith et al., 1999). Wright et al. (2013) suggested that the scenario methods are effective in a variety of fields including human-robot (or human-computer) interaction studies (Friedman, 1995; Leo and Huh, 2020; Furlough et al., 2021). In each scenario of this study, human and robot agents succeed or slightly fail at a task in a service or expert situation. The reason why we used minor failure scenarios rather than major incidents is that the robots entering the public will be tested for performance in advance, and minor failures are more likely to occur in reality.

## 2 Experiment 1

Experiment 1 was approved by the Institutional Review Board of Graduate School of Education, Kyoto University (CPE-557).

### 2.1 Method

#### 2.1.1 Participants

We recruited 80 participants (mean age = 42.8, *SD* = 7.8, range = 24–67 years; 56 male and 24 female) using the crowdsourcing site (Lancers, http://www.lancers.jp). The sample size was calculated using PANGEA (v0.2) (Westfall, 2015), with the within-participant factorial design, politeness (polite vs. casual) × service agent (robot vs. human) × service type (service vs. expert), a medium effect size of .25 (Cohen, 1988), and the appropriate sample size to ensure power = .80 at a significance level of 5%. The required sample size was 65, and this number of participants ensured power = .80 not only for the interaction of the three factors but also for the main effect of each factor. Considering the possibility that some people may have to be excluded due to the attention check, we decided to obtain 80 samples before beginning the data collection. However, since none of the participants had incomplete data and all passed the attention check, we analyzed all the data. All participants provided informed consent prior to the experiments.

#### 2.1.2 Stimuli

The stimuli included 32 scenarios including four basic (two for each service type scene; service vs. expert) and eight variations (politeness: polite vs. casual × service agent: robot vs. human × success of the task: success vs. minor failure) for each (the summary of the variation of scenarios is shown in Table 1). An example of scenario (polite, robot, success in service) is below.

*You have come to a department store to purchase a birthday present. This department store has an android-type robot installed at the reception desk. You ask at the reception where you could purchase a leather bag. The reception robot looks at you and bows while saying, “Welcome to our department store. How may I help you, sir (or ma’am)?” Then, the robot approaches you and explains with a gesture, saying, “If you would like to browse leather bags, you should go to Shop A. If you proceed straight down that aisle, turn right at the end, and go straight for a while, you will find Shop A on your right,” pointing in the direction of the destination. And, the robot adds “I hope you are able to find a good bag.” You proceed straight in the direction shown by the robot, turn right at the end, and go straight.*

*You successfully find shop a on your right.*

**TABLE 1:** The summary of variations of scenarios.

Success of the task	Politeness	Service agent	Service type	Job	Situation
Success	Polite	Human	Service	Reception Clerk	Guidance to stores in department store
				Sales Clerk	Instructions for sending forms at department stores
			Expert	Nursing Staff	Guidance to the examination room at a general hospital
				Rehabilitation Doctor	Instructions for rehabilitation of back pain
		Robot	Service	Reception Clerk	Guidance to stores in department store
				Sales Clerk	Instructions for sending forms at department stores
			Expert	Nursing Robot	Guidance to the examination room at a general hospital
				Rehabilitation Robot	Instructions for rehabilitation of back pain
	Casual	Human	Service	Reception Clerk	Guidance to stores in department store
				Sales Clerk	Instructions for sending forms at department stores
			Expert	Nursing Staff	Guidance to the examination room at a general hospital
				Rehabilitation Doctor	Instructions for rehabilitation of back pain
		Robot	Service	Reception Clerk	Guidance to stores in department store
				Sales Clerk	Instructions for sending forms at department stores
			Expert	Nursing Robot	Guidance to the examination room at a general hospital
				Rehabilitation Robot	Instructions for rehabilitation of back pain
Minor failure	Polite	Human	Service	Reception Clerk	Guidance to stores in department store
				Sales Clerk	Instructions for sending forms at department stores
			Expert	Nursing Staff	Guidance to the examination room at a general hospital
				Rehabilitation Doctor	Instructions for rehabilitation of back pain
		Robot	Service	Reception Clerk	Guidance to stores in department store
				Sales Clerk	Instructions for sending forms at department stores
			Expert	Nursing Staff	Guidance to the examination room at a general hospital
				Rehabilitation Robot	Instructions for rehabilitation of back pain
	Casual	Human	Service	Reception Clerk	Guidance to stores in department store
				Sales Clerk	Instructions for sending forms at department stores
			Expert	Nursing Staff	Guidance to the examination room at a general hospital
				Rehabilitation Doctor	Instructions for rehabilitation of back pain
		Robot	Service	Reception Clerk	Guidance to stores in department store
				Sales Clerk	Instructions for sending forms at department stores
			Expert	Nursing Staff	Guidance to the examination room at a general hospital
				Rehabilitation Robot	Instructions for rehabilitation of back pain

Note. in minor failure scenarios, it is implied that following the robot’s instructions did not solve the problem and the person had to deal with them a bit but did not provide details regarding the failure.

Other scenarios are shown in the Supplementary Appendix. All^1^ scenarios were presented in Japanese, each with the following questions: “How much do you like this robot/human (=preference),” “Would you like to interact with this robot/human again (= future interaction motivation),” “How much do you think this robot/human helps you accomplish the task (= contribution to achieving a goal).” They were rated on a 7-point Likert scale (1 = strongly disagree, 7 = strongly agree).

In the preliminary experiment, we confirmed that the scenarios had different impressions of politeness. Thirty participants (mean age = 42.9, *SD* = 7.4, range = 31–62; 23 male and 7 female) in Lancers, who were different from those who participated in Experiment 1, evaluated the politeness of agents in each scenario based on a question, “How polite do you think this robot/human is (=politeness)” rated on a 7-point Likert scale (1 = strongly disagree, 7 = strongly agree). A 2 (politeness vs. casual) × 2 (robot vs. human) × 2 (service vs. expert) repeated-measures analysis of variance (ANOVA) revealed that the main effect of politeness was significant both in success situations, *F* (1, 29) = 116.43, *p* < .001, ɳ_p_^2^ = .80, and in minor failure situations, *F* (1, 29) = 93.78, *p* < .001, ɳ_p_^2^ = .76, indicating that polite scenarios were evaluated as more polite than were casual scenarios. Furthermore, to check the manipulation, we compared polite and casual scenarios with other identical factors using paired *t*-tests, and found that in all situations, the polite scenarios was judged to be more polite than were the casual scenarios (*p*s < 0.001).

#### 2.1.3 Procedure

**FIGURE 1:**
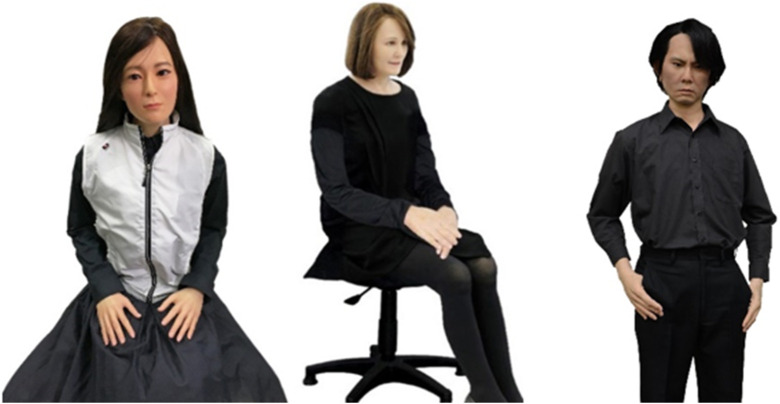
Sample images of android robots which were presented to participants. Note that this experiment was conducted online; therefore, the image size was dependent on the participant’s display.

Participants accessed the web page created by Qualtrics via their own computers or tablets to participate in the experiment. After signing the informed consent forms, they were provided the following instruction with three examples of android robot pictures in the ABOT (Anthropomorphic roBOT) Database (Phillips et al., 2018) (ABOT ID; 196, 198, and 239, see Figure 1):

You will be provided a series of scenarios that you may encounter in your life. Read them carefully. Some scenarios may seem similar, but they differ in important aspects. Please understand that some scenarios may seem beyond today’s technology, but this is because we are interested in your thoughts about situations you may encounter in real life in the future.

The android robots in *the* scenarios indicate robots having a head, a torso, two arms, two legs, and a physical appearance similar to that of humans, including a face. These robots can speak, make facial expressions, and perform actions just like humans. It is expected that smooth, more human-like movements will be possible in the future. Android robots currently under development are shown here, so please read the following scenarios with reference to them.

Android robots were chosen as the target in this study because people are difficult to imagine robots with a mechanical appearance interacting in the same way as humans in society, and are easy to imagine robots with a human-like appearance in the future society. As the scenario experiment required participants’ imagination, we presented images of robots to help them imagine their real-life existence. Sample images of android robots were chosen based on the criteria that they should wear regular clothes (not dresses), show the entire body, and include a male robot.

Next, 32 scenarios were presented in random order, and participants evaluated the human/robot agent in each scenario. The experiment was conducted at the participants’ own pace. After evaluating all the scenarios, an attention check, similar to the one employed in a previous scenario study (Gawronski et al., 2017), was conducted. Finally, the participants answered questions concerning their age and gender. They then received JPY 500 as compensation.

#### 2.1.4 Pre-processing and analysis

All analyses were conducted using Python 3.9.1 and R 4.2.3. Since the decision criteria were expected to differ between when a task goal was achieved and when minor failures occurred based on previous studies (Inbar and Meyer, 2019; Leo and Huh, 2020), the task success and minor failure situations were separately analyzed before beginning the analysis (i.e., planned comparison).

### 2.2 Results

Table 2 shows descriptive information in minor failure and success scenarios. In success/minor failure situations, we separately performed a 2 (politeness: politeness vs. casual) × 2 (service agent: robot vs. human) × 2 (service type: service vs. expert) within ANOVA on evaluations concerning preference, future interaction motivations, and contribution to achieving a goal.

**TABLE 2:** Descriptive statistics in minor failure and success scenarios in experiment 1.

Success of the task	Politeness	Service agent	Service type	Preference	Future interaction motivation	Contribution
				M (SD)	M (SD)	M (SD)
Minor failure	Polite	Human	Service	4.11 (1.12)	3.43 (1.32)	2.94 (1.36)
			Expert	4.27 (1.00)	3.78 (1.16)	3.64 (1.29)
		Robot	Service	4.01 (1.18)	3.35 (1.29)	2.90 (1.31)
			Expert	4.15 (1.01)	3.70 (1.09)	3.51 (1.26)
	Casual	Human	Service	3.20 (1.09)	2.58 (1.04)	2.52 (1.10)
			Expert	3.67 (0.93)	3.41 (1.05)	3.56 (1.23)
		Robot	Service	3.30 (1.17)	2.66 (1.18)	2.59 (1.22)
			Expert	3.70 (0.94)	3.31 (1.01)	3.49 (1.24)
Success	Polite	Human	Service	5.93 (0.92)	6.16 (0.89)	6.55 (0.69)
			Expert	5.65 (0.87)	5.98 (0.83)	6.40 (0.69)
		Robot	Service	5.92 (0.92)	6.15 (0.84)	6.59 (0.59)
			Expert	5.64 (0.86)	5.89 (0.92)	6.38 (0.72)
	Casual	Human	Service	4.92 (0.86)	5.22 (0.97)	6.34 (0.71)
			Expert	4.86 (0.86)	5.30 (0.91)	6.14 (0.84)
		Robot	Service	4.93 (0.83)	5.33 (0.87)	6.33 (0.72)
			Expert	5.02 (0.78)	5.43 (0.79)	6.22 (0.77)

#### 2.2.1 Minor failure situations

**FIGURE 2:**
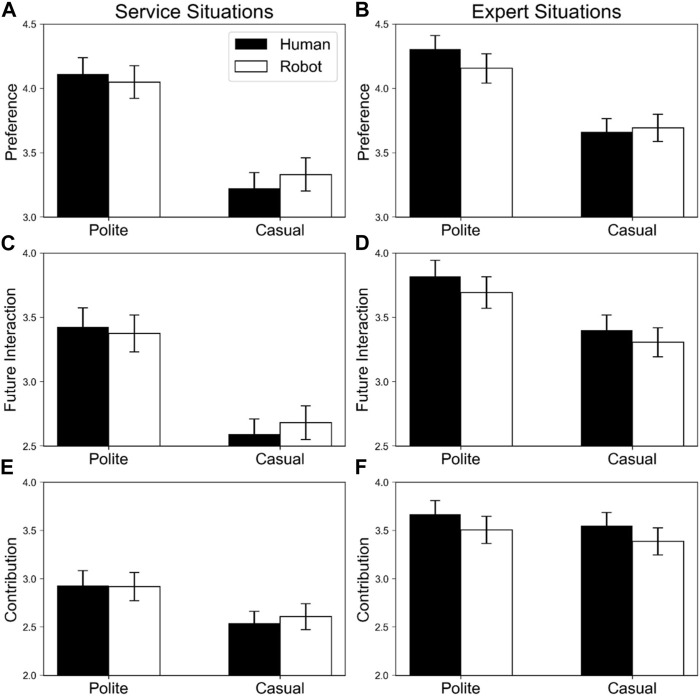
Means and standard deviations of evaluations for robot and human agents in minor failure scenarios. The black and white bars indicate human and robot agents, respectively. Error bars indicate standard errors. Panels **(A,B)** display preferences in service and expert situations respectively; **(C,D)** indicate future interaction motivation in service and expert situations, respectively; and **(E,F)** display contribution to achieving a goal in service and expert situations, respectively.

Figure 2 shows evaluations for robot and human agents in minor failure scenarios.

##### 2.2.1.1 Preference

Significant main effects of politeness, *F* (1, 79) = 60.60, *p* < .001, η_p_^2^ = .43, and service type, *F* (1,79) = 20.11, *p* < .001, η_p_^2^ = .20 were observed, indicating that polite agents were preferred over casual agents, and agents in expert situations were preferred over those in service situations.

In addition, significant interaction between politeness and service type was also observed, *F* (1,79) = 9.63, *p* = .003, η_p_^2^ = .11. Follow-up analysis indicated that simple main effects of politeness were significant in both service and expert situations, *F* (1, 79) = 53.31, *p* < .001, η_p_^2^ = .40, and *F* (1, 79) = 48.39, *p* < .001, η_p_^2^ = .38, respectively, indicating that polite agents were preferred over casual agents in both situations, and the difference of politeness was smaller in expert situations than in service situations.

Furthermore, the interaction between politeness and service agent was also significant, *F* (1, 79) = 5.22, *p* = .025, η_p_^2^ = .06. Follow-up analysis indicated that simple main effects of politeness were significant in both robot and human agents, *F* (1, 79) = 41.67, *p* < .001, η_p_^2^ = .35, and *F* (1, 79) = 60.20, *p* < .001, η_p_^2^ = .43, respectively. In other words, polite agents were preferred over casual agents as humans and robots, and the difference in politeness was greater in human agents than in robot agents.

Other effects including the three-way interaction were not significant (*F*s < 9.63, *p*s > .32).

##### 2.2.1.2 Future interaction motivation

Significant main effects of politeness, *F* (1, 79) = 52.08, *p* < .001, η_p_^2^ = .40, and service type, *F* (1, 79) = 63.47, *p* < .001, η_p_^2^ = .45, were observed, indicating that participants wanted to interact more with polite agents and agents in expert situations than with casual agents and agents in service situations.

Interaction between politeness and service type was also significant, *F* (1, 79) = 16.50, *p* < .001, η_p_^2^ = .17. Follow-up analysis indicated that simple main effects of politeness were significant in both service and expert situations, *F* (1, 79) = 59.76, *p* < .001, η_p_^2^ = .43, and *F* (1, 79) = 22.48, *p* < .001, η_p_^2^ = .22, respectively. This revealed that participants wanted to interact more with polite agents than with casual agents in both situations, and the difference of politeness was smaller in expert situations than in service situations.

Other effects were not significant (*F*s < 1.93, *p*s > .17).

##### 2.2.1.3 Contribution

Significant main effects of politeness, *F* (1, 79) = 22.74, *p* < .001, η_p_^2^ = .22, and service type, *F* (1, 79) = 99.69, *p* < .001, η_p_^2^ = .56 were observed, indicating that polite agents were perceived to contribute more than were casual agents; expert situations were also perceived to contribute more than were service situations.

The interaction between politeness and service type was significant, *F* (1, 79) = 9.16, *p* = .003, η_p_^2^ = .10. Follow-up analysis indicated that simple main effects of politeness were significant in both service and expert situations, *F* (1, 79) = 25.77, *p* < .001, η_p_^2^ = .25, and *F* (1, 79) = 4.63, *p* = .035, η_p_^2^ = .06, respectively. In other words, polite agents were perceived to contribute more than were casual agents in both situations, and the difference in politeness was smaller in expert than in service situations.

Furthermore, the interaction between service agent and service type was also significant, *F* (1, 79) = 5.02, *p* = .028, η_p_^2^ = .06. Follow-up analysis indicated that a simple main effect of agent was significant in expert situations, *F* (1, 79) = 6.95, *p* = .010, η_p_^2^ = .00, but not in service situations, *F* (1, 79) = 0.21, *p* = .651, η_p_^2^ = .08. In other words, humans were perceived to contribute more than robots in expert situations, but the contributions were equivalent in service situations.

Other effects were not significant (*F*s < 2.25, *p*s > .14).

#### 2.2.2 Success situations

**FIGURE 3:**
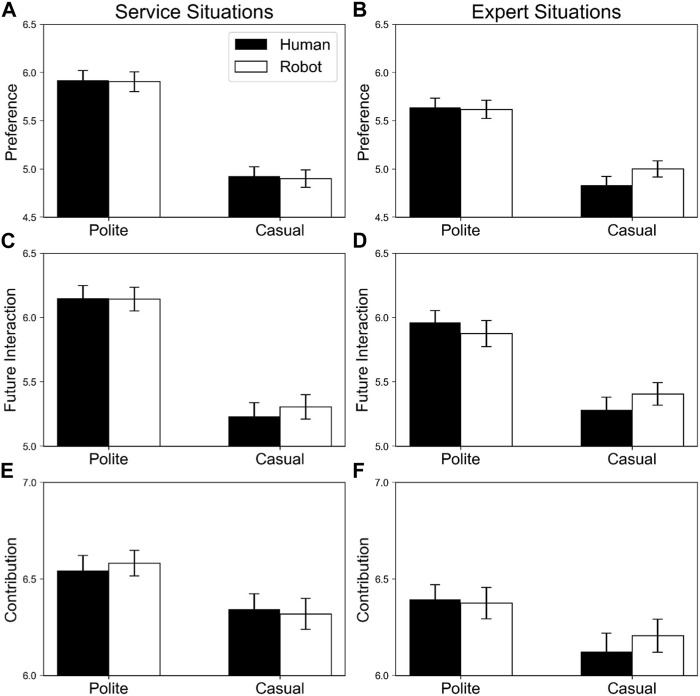
Means and standard deviations of evaluations for robot and human agents in success scenarios. The black and white bars indicate human and robot agents, respectively. Error bars indicate standard errors. Panels **(A,B)** display preference in service and expert situations, respectively, **(C,D)** indicate future interaction motivation in service and expert situations, respectively; and **(E,F)** display contribution to achieving a goal in service and expert situations, respectively.

Figure 3 depicts the evaluations for agents in success scenarios.

##### 2.2.2.1 Preference

Significant main effects of politeness, *F* (1, 79) = 100.93, *p* < .001, η_p_^2^ = .56, and service type, *F* (1, 79) = 11.07, *p* = .001, η_p_^2^ = .12, and interactions between politeness and service type, *F* (1, 79) = 14.36, *p* < .001, η_p_^2^ = .15, and between politeness, service agent, and service type, *F* (1, 79) = 4.53, *p* = .036, η_p_^2^ = .05 were observed.

To conduct follow-up analysis of the three-way interaction, data were divided into two groups according to the level of service type. For the service situation, a significant main effect of politeness, *F* (1, 79) = 100.12, *p* < .001, η_p_^2^ = .56, was observed indicating that polite situations were preferred over casual ones. Other effects were not significant (*F*s < 0.15, *p*s > .70). For the expert situation, a significant main effect of politeness, *F* (1, 79) = 68.43, *p* < .001, η_p_^2^ = .46, was observed, indicating that polite situations were preferred over casual ones, and interaction between politeness and service agent, *F* (1, 79) = 5.03, *p* = .028 η_p_^2^ = .06 was also observed. Further analysis indicated that simple main effects of politeness were significant in both human and robot agents, *F* (1, 79) = 68.76, *p* < .001, η_p_^2^ = .47, and *F* (1, 79) = 43.11, *p* < .001, η_p_^2^ = .35, respectively. The results revealed that polite situations were preferred over casual situations both in human and robot agents, and the difference in politeness was greater in human than in robot agents in expert situations.

##### 2.2.2.2 Future interaction motivation

A significant main effect of politeness, *F* (1, 79) = 95.23, *p* < .001, η_p_^2^ = .55 was observed, indicating that participants were more likely to interact with polite agents than with casual agents.

The interaction between politeness and service type was also significant, *F* (1, 79) = 11.98, *p* < .001, η_p_^2^ = .13. Follow-up analysis indicated that simple main effects of politeness were significant in both service and expert situations, *F* (1, 79) = 101.80, *p* < .001, η_p_^2^ = .56, and *F* (1, 79) = 45.01, *p* < .001, η_p_^2^ = .36, respectively. Thus, participants were more likely to interact with polite than casual agents in both situations, and the difference in politeness was smaller in expert than in service situations.

Other effects were not significant (*F*s < 2.62, *p*s > .11).

##### 2.2.2.3 Contribution

Significant main effects of politeness, *F* (1, 79) = 37.04, *p* < .001, η_p_^2^ = .32, and service type, *F* (1, 79) = 12.70, *p* = .001, η_p_^2^ = .14, and three-way interaction between politeness, service agent, and service type, *F* (1, 79) = 3.98, *p* = .049, η_p_^2^ = .05, were observed.

For further analysis of the three-way interaction, the data were divided into two groups according to the level of service type. For both the service and expert situations, significant main effects of politeness were observed, *F* (1, 79) = 26.83, *p* < .001, η_p_^2^ = .25, and *F* (1, 79) = 20.97, *p* < .001, η_p_^2^ = .21, respectively. Other effects were not significant in either situation, *F*s < 1.29, *p*s > .26, and *F*s < 1.75, *p*s > .19, respectively. The results indicate that polite agents were perceived to contribute more than did casual agents.

### 2.3 Discussion

Experiment 1 examined differences in impressions between robots and humans who succeeded or slightly failed at tasks with the same speech and actions. People preferred and were more willing to interact with polite agents, as they believed that such agents helped them accomplish the task regardless of whether the task was a success. The results concerning preference were consistent with previous studies, which reveal that polite robots were preferred in both service and expert situations when the task was a success (Inbar and Meyer, 2019; Rea et al., 2021). Moreover, our results provide novel indication that in expert situations, people were willing to interact again with polite robots even when they did a minor failure in a task.

Although in both service and expert situations, participants preferred polite agents over casual ones and were willing to interact with them again, the effect of politeness was larger in service situations than in expert situations, suggesting that participants considered politeness to be more important in service situations than in expert situations. Even when the task failed, polite agents were perceived to contribute more to accomplishing the task than were casual agents in service and expert situations. These findings are consistent with previous studies showing that politeness was involved in positive evaluations of interaction in service situations (Inbar and Meyer, 2019) and we found that this can be extended to expert scenarios.

Regardless of the service type, both human and robot polite agents were preferred over casual agents, but the difference between polite and casual agents was larger among humans than among robots. Politeness is an important factor in interaction with robot agents, albeit not as important as with human agents.

Experiment 1 revealed that when there were minor failures in tasks, the politeness of robot agents affected participants’ preference less than did the politeness of human agents. However, for future interaction motivations and perceptions of contributions of the agent, politeness had the same effect across robot and human agents. Why are people willing to interact again with polite robots that failed to accomplish tasks despite their low preference toward them? One hypothesis is that politeness affects trustworthiness of agents, an unfavorable result to be attributed to an accidental occurrence. Although robots are generally considered to have high replicability in their actions (i.e., stable performance), this perspective may lead people to believe that they will not make the same mistakes again. To examine this, in Experiment 2, we investigated people’s beliefs about agents regarding replicability and expectations for better results using the same scenarios.

## 3 Experiment 2

Experiment 2 was approved by the Institutional Review Board of Graduate School of Education, Kyoto University (CPE-562).

### 3.1 Method

#### 3.1.1 Participants

We recruited 80 participants (mean age = 43.6, *SD* = 11.3, range = 22–76 years; 52 male and 28 female) using the crowdsourcing site Lancers. The sample size was calculated using the same method as that in Experiment 1. None of the participants of Experiment 1 participated in Experiment 2, and provided informed consent prior to the experiment.

#### 3.1.2 Stimuli

The stimuli were identical to Experiment 1.

#### 3.1.3 Procedure

All procedures were the same as those in Experiment 1 except for the questions presented after each scenario. The three questions (preference, future interaction motivations, and contribution to achieving a goal) in Experiment 1 were replaced by the following two questions: “How likely are you to get a similar result when you interact with this person/robot again? (=replicability),” “How likely are you to get a better result when you make a similar request to another person/robot in another department store/hospital? (= expectations for better results)” with a 7-point Likert Scale (1 = not agree, 7 = agree).

#### 3.1.4 Pre-processing and analysis

Success/minor failure situations were separately analyzed as in Experiment 1.

### 3.2 Results

Table 3 shows descriptive information in minor failure and success scenarios. In success/minor failure situations, we separately performed a 2 (politeness: politeness vs. casual) × 2 (service agent: robot vs. human) × 2 (service type: service vs. expert) within ANOVA on the evaluations concerning replicability and expectations for better results to achieving a goal.

**TABLE 3:** Descriptive statistics in minor failure and success scenarios in experiment 2.

Success of the task	Politeness	Service agent	Service type	Replicability	Expectations for better results
				M (SD)	M (SD)
Minor failure	Polite	Human	Service	4.16 (1.19)	5.66 (1.02)
			Expert	4.78 (1.12)	5.21 (0.10)
		Robot	Service	4.83 (1.33)	5.27 (1.11)
			Expert	5.25 (1.08)	4.88 (1.06)
	Casual	Human	Service	4.28 (1.30)	5.71 (0.99)
			Expert	4.73 (1.09)	5.45 (0.81)
		Robot	Service	5.04 (1.43)	5.28 (1.17)
			Expert	5.29 (1.22)	5.06 (1.09)
Success	Polite	Human	Service	6.19 (0.88)	3.81 (1.27)
			Expert	6.15 (0.70)	3.91 (1.15)
		Robot	Service	6.28 (0.80)	3.69 (1.21)
			Expert	6.12 (0.76)	3.81 (1.14)
	Casual	Human	Service	6.21 (0.67)	4.28 (1.13)
			Expert	6.02 (0.83)	4.10 (1.02)
		Robot	Service	6.23 (0.77)	3.98 (1.19)
			Expert	6.08 (0.83)	4.01 (0.98)

#### 3.2.1 Minor failure situations

**FIGURE 4:**
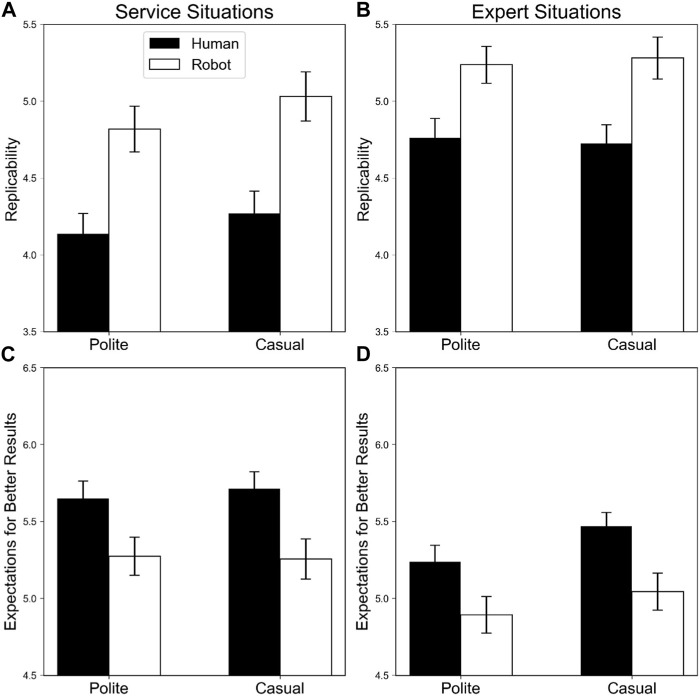
Means and standard deviations of replicability and expectations for better results to achieve a goal for robot and human agents in minor failure scenarios. The black and white bars indicate human and robot agents, respectively. Error bars indicate standard errors. Panels **(A,B)** display replicability in service and expert situations, respectively; **(C,D)** indicate expectations for better results in service and expert situations, respectively.

Figure 4 shows replicability and expectations for better results to achieve a goal for robot and human agents in minor failure scenarios.

##### 3.2.1.1 Replicability

Significant main effects of service agent, *F* (1, 79) = 25.86, *p* < .001, η_p_^2^ = .25, and service type, *F* (1, 79) = 36.31, *p* < .001, η_p_^2^ = .31 were observed, indicating that robot agents were more likely than human agents to produce the same results, and agents were more likely to replicate the results in expert situations than in service situations.

Other main effects and interactions were not significant (*F*s < 3.52, *p*s > .06).

##### 3.2.1.2 Expectations for better results

Significant main effects of politeness, *F* (1, 79) = 6.94, *p* = .010, η_p_^2^ = .08, service agent, *F* (1, 79) = 22.85, *p* < .001, η_p_^2^ = .22, and service type, *F* (1, 79) = 18.60, *p* < .001, η_p_^2^ = .19, were observed, indicating that expectations for better results from interacting with other agents were higher in casual and human agents than in polite and robot agents. Moreover, participants expected better results with other agents in service than in expert situations.

Interactions were not significant (*F*s < 3.29, *p*s > .74).

#### 3.2.2 Success situations

**FIGURE 5:**
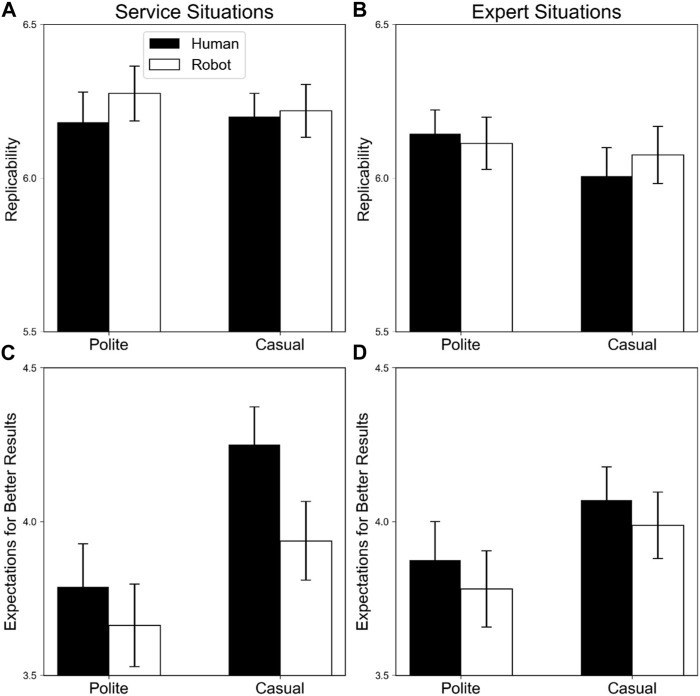
Means and standard deviations of replicability and expectations for better results to achieving a goal for robot and human agents in success scenarios. The black and white bars indicate human and robot agents, respectively. Error bars indicate standard errors. Panels **(A,B)** display replicability in service and expert situations, respectively; **(C,D)** indicate expectations for better results in service and expert situations, respectively.

Figure 5 depicts the replicability and expectations for better results to achieving a goal for robot and human agents in success scenarios.

##### 3.2.2.1 Replicability

Significant main effects of politeness, *F* (1, 79) = 4.33, *p* = .041, η_p_^2^ = .05, and service type, *F* (1, 79) = 13.39, *p* = .001, η_p_^2^ = .14 were observed, indicating that polite agents were more likely than casual agents to produce the same results, and agents were more likely to replicate the results in service situations than in expert situations.

Other effects were not significant (*F*s < 2.72, *p*s > .10).

##### 3.2.2.2 Expectations for better results

Significant main effects of politeness, *F* (1, 79) = 19.56, *p* < .001, η_p_^2^ = .20, and service agent, *F* (1, 79) = 6.94, *p* = .010, η_p_^2^ = .08 were observed, indicating that expectations for better results from interacting with other agents were higher in casual and human agents than in polite and robot agents, respectively.

Other effects were not significant (*F*s < 3.05, *p*s > .08).

### 3.3 Discussion

Experiment 2 revealed that politeness affected perceived replicability and expectations for better results. In task success situations, polite agents were considered to be able to replicate the results, whereas in task failure situations, they were not. Moreover, regardless of task success/minor failure, polite agents lowered people’s expectations of better results from other agents. These findings suggest that polite robots are considered to replicate successes better than casual robots, but not necessarily to replicate failure more often.

People believed that minor failures in expert situations were more likely to be repeated but less expected for better results compared with service situations. People also believed that robot agents may replicate minor task failures, and their expectations for better results from other agents was lower than those from human agents. Since both experts and robots can provide services by consistently demonstrating the same performance, participants believe that they would reproduce the same results.

## 4 General discussion

In this study, we aimed to identify the effects of politeness and service type on robot and human agents’ preference, future interaction motivation, and contribution to achieving a goal. We also examined the replicability and expectations for better results of each agent in service and expert situations with task success and minor failure. Through two experiments, we found that, regardless of robot and human agents, polite behaviors when compared to casual behaviors were evaluated higher, indicating higher preference, future interaction motivation, perceived contribution, and perceived replicability of task success, and with lower expectation of better results from other agents. These results suggest that politeness design of robotics plays an important role in encouraging people to accept the robot as well as human agents and accelerate human-robot interaction in society.

When agents slightly failed in their task (e.g., misdirection of a room or rehabilitation amount), politeness was less of an issue for preference of robot agents than that of human agents, but there the same effect of politeness for robot and human agents was observed in terms of future interaction motivation and contribution to achieving a goal. One reason for people’s low preference of and willingness to interact again with polite robots despite minor failures in a task may be that people do not expect other agents to have better results. Thus, politeness influences the motivation to interact again with robots and humans, rather than preference. Another possible explanation is that since people have emphasized the contribution of polite versus casual agents, it may lead to a motivation to interact again, regardless of preference for agent, which suggests that politeness design could encourage further interactions with robots in multiple aspects.

People believed that experts were more replicable, and had lower expectations of better results from other agents in expert contexts compared with general service contexts in minor failure situations. The finding that politeness was less of a concern in expert situations than in general service situations (Experiment 1 in this study, Inbar and Meyer, 2019; Rea et al., 2021) indicates that the robot design of politeness should be more effective in general service situations than in expert situations.

As studies proposing personalize agents’ politeness levels according to each participant (Firdaus et al., 2022; Smith et al., 2022), participants’ demographic differences (e.g., age and gender) may affect politeness impressions (Jucks et al., 2018; Kumar et al., 2022; Miyamoto et al., 2019; Rana et al., 2021; Zhu and Kaber, 2012, but not observed in Inbar and Meyer, 2015; Inbar and Meyer, 2019; Lee et al., 2019). As this study did not aim to investigate demographic differences, we did not control the numbers of male and female participants and participants’ ages, but conducted *a posteriori* analysis regarding gender differences. The results showed no significant gender differences for most dependent variables, and significant differences that were found were inconsistent across task-success and -failure scenarios. Therefore, the results of this study do not provide convincing evidence for gender differences in politeness impressions of service agents (Inbar and Meyer, 2015; 2019; Lee et al., 2019).

Although this study investigated the effect of politeness design on robot impressions in interactions, future research should examine the relationship between the prior robot interaction impressions and the effect of politeness design. People form impressions of robots at a glance (Prakash and Rogers, 2015), which is likely to be updated during actual interactions. Therefore, the effect of polite behavior differs depending on the impressions people have. If people have a positive impression of robots, somewhat casual behaviors may be appreciated; conversely, if they have an unfavorable impression, even polite behaviors may be ineffective when they fail in their tasks. Such an extension would make it possible to find effective designs that make robots acceptable to people, including attitude (i.e., how to speak and behave) and appearance.

Although previous studies pointed that pre-attitudes toward robots affect robot impressions (e.g., Nomura et al., 2007), it is more important to consider robot designs that are acceptable regardless of pre-attitude to promote introducing robots into society than those that depend on pre-attitude. It is possible that pre-attitudes toward robots may influence impression formation based on interactions with polite robots, but this is a topic for future research. Note that in our previous study, we found that impressions formed from interactions are independent—at least—of the attitude induced by their appearances (Saeki and Ueda, 2024).

As described in the introduction, the experiments were scenario-based because of the ease of manipulating factors without causing actual failures. However, the reality of the situation is also an important factor that affects robot impressions in human-robot interactions, and a few studies examined the impact of robot failures in real-life. Gideoni et al. (2022) found that the higher the personal relevance of the failure, the greater the decrease in the likability and willingness to further use robots in human-robot collaboration tasks. Although this study was not concerned to human-robot collaborations or higher personal-relevance failures, the reduced intention for future interactions after a failure was similar to Gideoni et al. (2022). Moreover, we showed that politeness may mitigate this decrement. In the future, extending this study to real-life interactions would provide more realistic findings.

This study provides evidence of the importance of politeness design on impressions of interactive agents. The effect of politeness design is critical for human rather than robot agents, but the directions of the effect are similar for both, indicating that polite agents are rated higher and people would like to interact with them again compared with casual agents. Thus, in numerous situations, the implementation of politeness would encourage people to accept the robot, thereby facilitating human-robot interactions in society.

## Data Availability

The original contributions presented in the study are included in the article/Supplementary Material, further inquiries can be directed to the corresponding authors.

## Footnotes

1 The scenarios have been translated into English for publication. We have confirmed that they have the same meaning as the Japanese ones presented in the experiment by back translation.

## References

[B1] Bitner M. J. Booms B. H. Tetreault M. S. (1990). The service encounter: diagnosing favorable and unfavorable incidents. J. Mark. 54, 71–84. 10.1177/002224299005400105

[B2] Brown S. W. Swartz T. A. (1989). A gap analysis of professional service quality. J. Mark. 53, 92–98. 10.1177/002224298905300207

[B3] Castro-González Á. Castillo J. C. Alonso-Martín F. Olortegui-Ortega O. V. González-Pacheco V. Malfaz M. (2016). “The effects of an impolite vs. a polite robot playing rock-paper-scissors,” in Social robotics: 8th international conference. ICSR 2016, Kansas city, MO, USA, proceedings. Editors Agah A. Cabibihan J. J. Howard A. Salichs M. He H., 8, 306–316. 10.1007/978-3-319-47437-3_30

[B4] Cohen J. (1988) Statistical power analysis for the behavioral sciences. 2nd ed. Hillsdale, NJ: Lawrence Erlbaum.

[B5] Coulter K. S. Coulter R. A. (2002). Determinants of trust in a service provider: the moderating role of length of relationship. J. Serv. Mark. 16, 35–50. 10.1108/08876040210419406

[B6] Edwards A. Edwards C. Westerman D. Spence P. R. (2019). Initial expectations, interactions, and beyond with social robots. Comput. Hum. Behav. 90, 308–314. 10.1016/j.chb.2018.08.042

[B7] Firdaus M. Shandilya A. Ekbal A. Bhattacharyya P. (2022). Being polite: modeling politeness variation in a personalized dialog agent. IEEE Trans. Comput. Soc. Syst. 10, 1455–1464. 10.1109/TCSS.2022.3182986

[B8] Friedman B. (1995). “‘It’s the computer’s fault’: reasoning about computers as moral agents,” in Conference companion on human factors in computing systems, 226–227. 10.1145/223355.223537

[B9] Furlough C. Stokes T. Gillan D. J. (2021). Attributing blame to robots: I. The influence of robot autonomy. Hum. Factors. 63, 592–602. 10.1177/0018720819880641 31613644

[B10] Gawronski B. Armstrong J. Conway P. Friesdorf R. Hütter M. (2017). Consequences, norms, and generalized inaction in moral dilemmas: the CNI model of moral decision-making. J. Pers. Soc. Psychol. 113, 343–376. 10.1037/pspa0000086 28816493

[B11] Gideoni R. Honig S. Oron-Gilad T. (2022). Is it personal? The impact of personally relevant robotic failures (PeRFs) on humans’ trust, likeability, and willingness to use the robot. Int J Soc Robotics, 1–19. 10.1007/s12369-022-00912-y PMC9452279 36097596

[B12] Hayes C. C. Miller C. A. (2016) Human-computer etiquette. Boca Raton, FL: CRC Press.

[B13] Inbar O. Meyer J. (2015). Manners matter: trust in robotic peacekeepers. Proc. Hum. factors ergonomics Soc. Annu. Meet. 59 (1), 185–189. 10.1177/1541931215591038

[B14] Inbar O. Meyer J. (2019). Politeness counts: perceptions of peacekeeping robots. IEEE Trans. Hum. Mach. Syst. 49, 232–240. 10.1109/THMS.2019.2900337

[B15] Jucks R. Linnemann G. A. Brummernhenrich B. (2018). Student evaluations of a (rude) spoken dialogue system insights from an experimental study. Adv. Hum. Comput. Interact. 2018, 1–10. 10.1155/2018/8406187

[B16] Kaiser F. G. Glatte K. Lauckner M. (2019). How to make nonhumanoid mobile robots more likable: employing kinesic courtesy cues to promote appreciation. Appl. Ergon. 78, 70–75. 10.1016/j.apergo.2019.02.004 31046961

[B17] Kumar S. Itzhak E. Edan Y. Nimrod G. Sarne-Fleischmann V. Tractinsky N. (2022). Politeness in human–robot interaction: a multi-experiment study with non-humanoid robots. Int. J. Soc. Robotics 14, 1805–1820. 10.1007/s12369-022-00911-z PMC9387416 35996386

[B18] Lee J.-G. Lee K. M. Ryu S.-H. (2019). Vehicle politeness in driving situations. Future Internet 11, 48. 10.3390/fi11020048

[B19] Lee N. Kim J. Kim E. Kwon O. (2017). The influence of politeness behavior on user compliance with social robots in a healthcare service setting. Int. J. Soc. Robot. 9, 727–743. 10.1007/s12369-017-0420-0

[B20] Leo X. Huh Y. E. (2020). Who gets the blame for service failures? Attribution of responsibility toward robot versus human service providers and service firms. Comput. Hum. Behav. 113, 106520. Art. number 106520. 10.1016/j.chb.2020.106520

[B21] Liberman-Pincu E. van Grondelle E. D. Oron-Gilad T. (2023). “Designing robots with the context in MindOne Design does not fit all,”. Human-friendly robotics 2022. HFR 2022. SPAR. Editors Borja P. Della Santina C. Peternel L. Torta E. (Cham: Springer), 26, 105–119. 10.1007/978-3-031-22731-8_8

[B22] Meyer J. Miller C. Hancock P. de Visser E. J. Dorneich M. (2016). Politeness in machine-human and human-human interaction. Proc. Hum. Factors Ergon. Soc. Annu. Meet. 60, 279–283. 10.1177/1541931213601064

[B23] Miyamoto T. Katagami D. Shigemitsu Y. Usami M. Tanaka T. Kanamori H. (2019). “Proposal of driving support agent which speak based on politeness theory,” in International conference on human–computer interaction. 235 (Cham: Springer). 10.1007/978-3-030-22666-4_17

[B24] Nomura T. Shintani T. Fujii K. Hokabe K. (2007). “Experimental investigation of relationships between anxiety, negative attitudes, and allowable distance of robots,” in Proceedings of the 2nd IASTED international conference on human computer interaction (Chamonix, France: ACTA Press), 13–18.

[B25] Phillips E. Zhao X. Ullman D. Malle B. F. (2018). What is humanlike? decomposing robot human-like appearance using the anthropomorphic roBOT (ABOT) database, in HRI ’18. Proc. 2018 ACM/IEEE Int. Conf. Human-Robot Interact., 105–113. 10.1145/3171221.3171268

[B26] Prakash A. Rogers W. A. (2015). Why some humanoid faces are perceived more positively than others: effects of human-likeness and task. Int. J. Soc. Robot. 7, 309–331. 10.1007/s12369-014-0269-4 26294936 PMC4539254

[B27] Rana K. Madaan R. Shukla J. (2021). “Effect of polite triggers in chatbot conversations on user experience across gender, age, and personality,” in 2021 30th IEEE international conference on robot & human interactive communication (RO-MAN), 813–819. 10.1109/RO-MAN50785.2021.9515528

[B28] Rea D. J. Schneider S. Kanda T. (2021). Is this all you can do? harder!’ the effects of (im) polite robot encouragement on exercise effort, in HRI ’21. Proc. 2021 ACM/IEEE Int. Conf. Human-Robot Interact., 225–233. 10.1145/3434073.3444660

[B29] Saeki W. Ueda Y. (2024). Sequential model based on human cognitive processing to robot acceptance. Front. Robot. Ai. 11, 1362044. 10.3389/frobt.2024.1362044 38560097 PMC10978770

[B30] Sinha P. K. Nanda R. S. McNeil D. W. (1996). Perceived orthodontist behaviors that predict patient satisfaction, orthodontist–patient relationship, and patient adherence in orthodontic treatment. Am. J. Orthod. Dentofac. Orthop. 110, 370–377. 10.1016/S0889-5406(96)70037-9 8876486

[B31] Smith A. K. Bolton R. N. (1998). An experimental investigation of customer reactions to service failure and recovery encounters: paradox or peril? J. Serv. Res. 1, 65–81. 10.1177/109467059800100106

[B32] Smith A. K. Bolton R. N. Wagner J. (1999). A model of customer satisfaction with service encounters involving failure and recovery. J. Mark. Res. 36, 356–372. 10.1177/002224379903600305

[B33] Smith C. Wen R. Elbeleidy S. Roy S. Williams T. Gorgemans C. (2022). Leveraging intentional factors and task context to predict linguistic norm adherence. Proc. Annu. Meet. Cognitive Sci. Soc. 44, 1962–1969.

[B34] Torrey C. Fussell S. R. Kiesler S. (2013). How a robot should give advice. 8th ACM/IEEE Int. Conf. Human-Robot Interact. (HRI) 2013, 275–282. 10.1109/HRI.2013.6483599

[B35] Urakami J. Sutthithatip S. (2021). Building a collaborative relationship between human and robot through verbal and non-verbal interaction. Companion 2021 ACM/IEEE Int. Conf. Human-Robot Interact., 257–261. 10.1145/3434074.3447171

[B36] Westfall J. (2015) PANGEA: power analysis for general ANOVA designs. Available at: http://jakewestfall.org/publications/pangea.pdf.4.

[B37] Westhoven M. Grinten T. Mueller S. (2019). “Perceptions of a help-requesting robot-effects of eye-expressions, colored lights and politeness of speech,” in Proceedings of Mensch und computer In Proceedings of Mensch und Computer, 43–54. 10.1145/3340764.3340783

[B38] Wright G. Cairns G. Bradfield R. (2013). Scenario methodology: new developments in theory and practice. Technol. Forecast. Soc. Change 80, 561–565. 10.1016/j.techfore.2012.11.011

[B39] Zhu B. Kaber D. (2012). Effects of etiquette strategy on human–robot interaction in a simulated medicine delivery task. Serv. Robot. 5 (3), 199–210. 10.1007/s11370-012-0113-3

