# Supplementary material for: Impact of politeness and performance quality of android robots on future interaction decisions: a conversational design perspective
Source: Front Robot AI. 2024 May 28;11:1393456. doi: 10.3389/frobt.2024.1393456 (PMC11165153; doi:10.3389/frobt.2024.1393456)
Supplement: Supplementary file 1 [file DataSheet1.docx]

**APPENDIX**

**Scenarios**

When the agent was a person, we presented the scenario that “an android-type robot” was replaced with “a person”.

***Polite, Service Scene***

**Guidance.** You have come to a department store to purchase a birthday present. This department store has an android-type robot installed at the reception desk. You ask at the reception where you could purchase a leather bag. The reception robot looks at you and bows while saying, “Welcome to our department store. How may I help you, sir (or ma’am)?” Then, the robot approaches you and explains with a gesture, saying, “If you would like to browse leather bags, you should go to Shop A. If you proceed straight down that aisle, turn right at the end, and go straight for a while, you will find Shop A on your right,” pointing in the direction of the destination. And, the robot adds “I hope you are able to find a good bag.” You proceed straight in the direction shown by the robot, turn right at the end, and go straight.

You successfully find Shop A on your right. (Success)

However, you find Shop A not on the right, but on the left side of the aisle. (Minor Failure)

**Instruction.** You have come to a department store to purchase a year-end gift. In this department store an android-type robot is in charge of part of customer service. You inform the robot salesclerk that you would like to send a year-end gift to your acquaintance. The robot salesclerk bows while saying, “Thank you for choosing our item, sir (or ma’am),” and continues, “I apologize for the trouble, but could I request that you write your acquaintance’s name and address on this slip?” pointing at each column on the slip one by one. You receive a ballpoint pen from the salesclerk, fill in the slip, and return it to the salesclerk, who then slightly bows while receiving the slip and says, “Thank you, sir (or ma’am). The gift will be delivered tomorrow. Please enjoy the rest of your shopping.”

You were successfully able to send your year-end gift. (Success)

However, the slip that the robot salesclerk gave you was the wrong slip and you had to rewrite it on a correct slip. (Minor Failure)

***Casual, Service Scene***

**Guidance.** You have come to a department store to purchase a birthday present. This department store has an android-type robot installed at the reception desk. You ask at the reception where you could purchase a leather bag. The reception robot keeps standing and says, “How can I help you?” Then the robot explains, saying, “You can find leather bags at Shop A. Go straight down that aisle, turn right at the end, go straight for a while, and you can find Shop A on your right,” pointing in the direction of the destination. You proceed straight in the direction shown by the robot, turn right at the end, and go straight.

You successfully find Shop A on your right. (Success)

However, you find Shop A not on the right, but on the left side of the aisle. (Minor Failure)

**Instruction.** You have come to a department store to purchase a year-end gift. In this department store an android-type robot is in charge of part of customer service. You inform the robot salesclerk that you would like to send a year-end gift to your acquaintance. The robot salesclerk says, “Thank you,” passes you a slip, and says, “Please write your acquaintance’s name and address on this slip.” You receive a ballpoint pen from the salesclerk, fill in the slip, and return it to the salesclerk, who then takes the slip and says, “The gift will be delivered tomorrow.”

You were successfully able to send your year-end gift. (Success)

However, the slip that the robot salesclerk gave you was the wrong slip and you had to rewrite it on a correct slip. (Minor Failure)

***Polite, Expert Scene***

**Guidance.** This is your first visit to this general hospital to receive a complete medical checkup. In this hospital an android-type robot is in charge of part of the nursing services. You complete one examination and have to move to another room to receive the next examination. The robot nurse looks at you and bows slightly, saying, “Thank you for your patience.” Then, the robot nurse approaches you and explains with a gesture, saying, “If you proceed straight down this hallway, turn right at the end and go straight for a while, you will find the Examination Room C on your right,” pointing in the direction of the destination. Then, you go straight in the direction the nurse indicated, turn right at the end, and go straight.

You successfully find Examination Room C on your right. (Success)

However, you find Examination Room C not on the right but on the left of the aisle. (Minor Failure)

**Instruction.** You developed lower back pain one year ago. The pain eased for a while, however it came back last week. Even though you took a painkiller, you still had the pain and suffered from it. To ease the pain, you began going to a clinic for rehabilitation. In this clinic an android-type robot is in charge of part of the rehabilitation service. The rehabilitation robot doctor listens to you, nodding his head and explains with a gesture, saying, “If you move your body like this, the pain will ease. So, please be sure to move your body like this at home.” When you return home from the clinic, you do the amount of rehabilitation exercise as instructed.

You realize after a little while that your lower back is better. (Success)

You realize after a little while that your lower back is not better. However, when you do more rehabilitation exercise than instructed, you realize that your lower back is better. (Minor Failure)

***Casual, Expert Scene***

**Guidance.** This is your first visit to this general hospital to receive a complete medical checkup. In this hospital an android-type robot is in charge of part of the nursing services. You complete one examination and have to move to another room to receive the next examination. The robot nurse says, “Go straight down this hallway, turn right at the end and go straight for a while, and Examination Room C is on your right,” pointing in the direction of the destination. Then, you go straight in the direction the nurse indicated, turn right at the end, and go straight.

You successfully find Examination Room C on your right. (Success)

However, you find Examination Room C not on the right but on the left of the aisle. (Minor Failure)

**Instruction.** You developed lower back pain one year ago. The pain eased for a while, however it came back last week. Even though you took a painkiller, you still had the pain and suffered from it. To ease the pain, you began going to a clinic for rehabilitation. In this clinic an android-type robot is in charge of part of the rehabilitation service. The rehabilitation robot doctor says, “Moving your body like this will ease the pain. So, be sure to move your body like this at home.” When you get back from the clinic, you do the instructed amount of rehabilitation exercise.

You realize after a little while that your lower back is better. (Success)

You realize after a little while that your lower back is not better. However, when you do more rehabilitation exercise than instructed, you realize that your lower back is better. (Minor Failure)
